# Insect biodiversity of the Algodones Dunes of California

**DOI:** 10.3897/BDJ.5.e21715

**Published:** 2017-11-24

**Authors:** Lynn S. Kimsey, Thomas J. Zavortink, Robert B. Kimsey, Steven L. Heydon

**Affiliations:** 1 University of California, Davis, United States of America

**Keywords:** Sonora, Mexico, Gran Desierto

## Abstract

Over a nine year period beginning in 2007 we surveyed the insects of the Algodones Dunes, Imperial Count, California, as part of a study undertaken for the U.S. Bureau of Land Management. In a series of 22 collecting trips ranging in duration from 2 to 8 days we thus far have accumulated records of 1,840 species, 21 orders and 244 families from the dunes. Hymenoptera constituted the most diverse order, comprising about 45% of all the species recovered. Insect diversity and abundance peaked during the hottest part of the year between the months of May and September. Life history traits of the insects sampled included herbivores (29.6%), parasitoids (28.7%), predators (18.1%), pollen/nectar feeders (10.9%), detritivores (6.2%) and scavengers (2.4%). Seventy-nine or 4% of the insect species collected in the dunes have been solely recorded from there, and 3% of the species almost certainly derive from adjacent aquatic habitats or agricultural ecosystems, as their life histories could not be completed in Algodones Dunes habitat. The insect fauna of the Algodones Dunes is unexpectedly rich and diverse.

## Introduction

The overall invertebrate biodiversity of any region in North America remains very poorly studied, and the proportional representation of life history traits of the most abundant group, insects, seems largely unknown. The Great Smoky Mountains National Park All Taxa Biological Inventory (Sharkey 2001) constitutes the most detailed such study, but most of the insect species level information in that study derived from target taxa, such as crane flies (Peterson et al. 2005) or ants (Lessard et al. 2007). No comprehensive inventories of insects from locations in the western U.S. or estimates of species richness exist. California encompasses a great diversity of climates, vegetation types, and invasive species, as well as direct and indirect human impacts, all of which affect insect diversity. With its diverse biomes, the California Floristic Province constitutes one of the world’s biodiversity hotspots (Myers et al. 2000). Although not part of the province, the southern California deserts may also have surprisingly rich insect diversity potentially with many endemic species.

**Figure 1.**
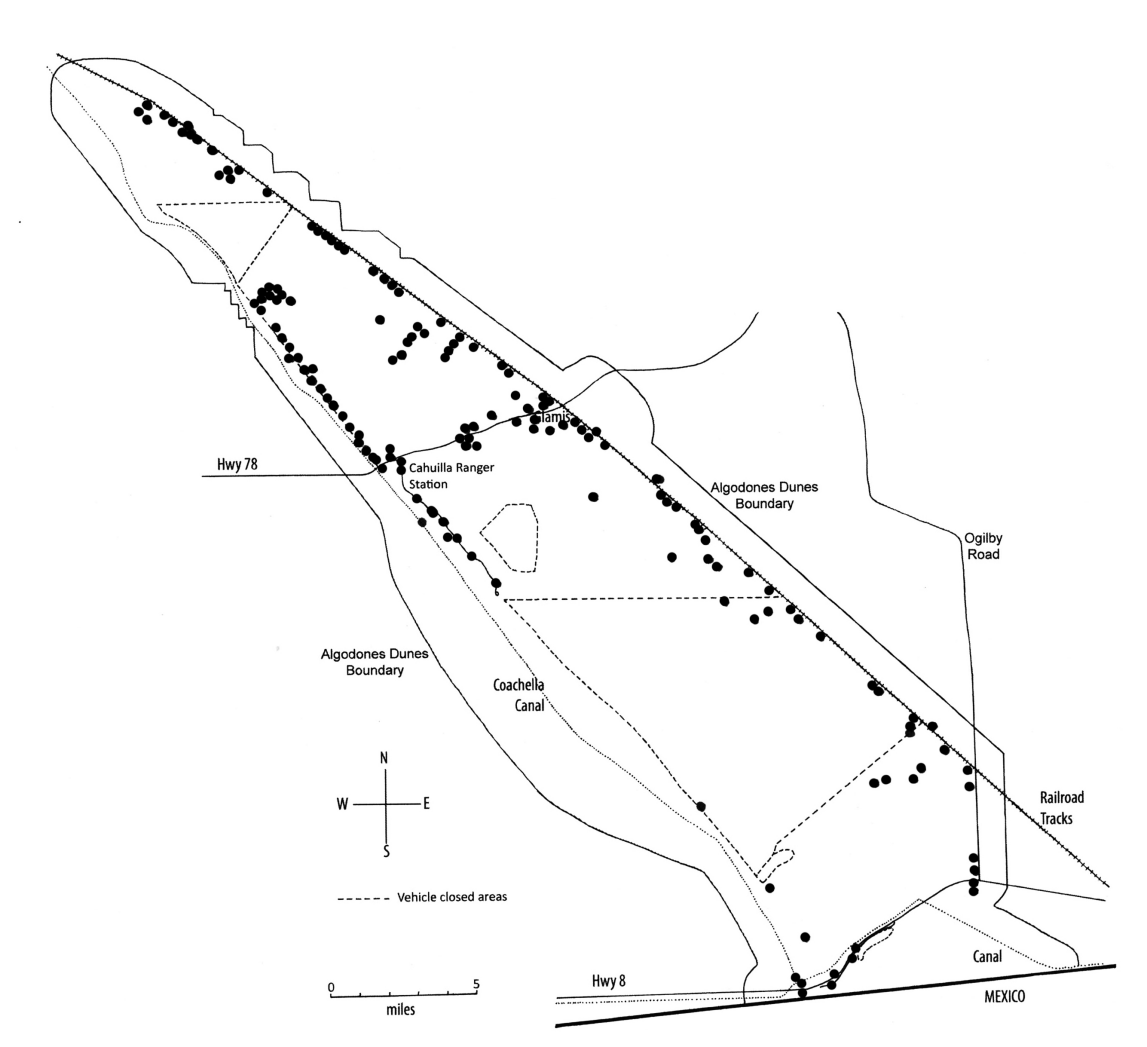
Map showing project collecting sites on the Algodones Dunes and the location of roads and canals.

The Algodones or Imperial Dunes constitute a major portion of the Imperial Sand Dunes Recreational Area (ISDRA), an extensive geographic feature in the southeastern corner of California. During the Pleistocene these dunes formed as part of a more extensive erg that extended from the southern end of the Salton Sea south through the Gran Desierto el Altar in Sonora, Mexico, to the Gulf of California. Periodic flooding and silt deposition by the Colorado River over thousands of years likely created the dunes (McCoy Jr et al. 1967, Merriam 1969). Today the dunes form an elongate band near the eastern margin of the Imperial Valley, narrowest to the north and broadest near the middle. They are roughly 64 km long and 10 km wide, and contain an estimated 10,760 million m^3^ of sand (McCoy Jr et al. 1967). Across the dunes’ southern edge a highway, a canal and a broad band of agricultural development along the Colorado River isolate them from the Gran Desierto to the south in Mexico (Fig. 1). Further north, California Highway 78 runs from east to west through the tiny community of Glamis, bisecting the dunes into northern and southern portions.

No natural surface water exists in these dunes, except for temporary pools caused by heavy rainfall run-off from the Chocolate and Cargo Muchacho Mountains. A diversity of manmade water resources on or adjacent to the dunes include wildlife “guzzlers” together with water delivery canals that run along the southern and western margins. Within the dunes the U.S. Bureau of Land Management maintains three such guzzlers, small in-ground water tanks for wildlife, primarily deer. The Coachella Canal runs along the western margin of the dunes, roughly 11 km west of Glamis. The All-American Canal transects the southern end of the dunes just above the Mexican border. The Union Pacific railroad runs the length of the eastern edge of the dunes. Where the tracks pass closest to the dunes Union Pacific drip irrigates rows of tamarisk trees as a windbreak to keep sand off the tracks (Fig. 2a). This irrigation forms small, semi-permanent saline pools beneath the trees. The Colorado River at its nearest point flows 6.5 km southeast of the Imperial Sand Dunes Recreation Area.

Humans have lived in or near the dunes for thousands of years and their influence on the biota of the dunes, minimal at first, has become more pronounced through time. Prehistoric Native American trails crossed the dunes along trade routes, which are followed today by sections of California Highway 78. The compacted soil of these trails is still visible in certain places paralleling the highway where it runs through a gap in the mountains east of the dunes. Native Americans also engaged in seasonal foraging on the dunes for food resources such as the elongate roots of sand food [*Pholisma sonorae* (Torr. ex Gray): (Boraginaceae)]. The mineral resources of the nearby mountains attracted European settlers to the area as early as the 1780’s creating towns and settlements. The largest of these towns was Tumco, which at its peak in the early 1900’s, was inhabited by over 3,000 people. The mine there closed permanently in 1942 and the site is now abandoned. The Algodones Dunes are now bordered on the south by the All-American Canal, and on the west by the Coachella Canal. These were constructed in the mid–30’s to provide irrigation to the Imperial Valley. During World War II, the dunes were a training ground for desert warfare troops and for air to ground combat, and spent ordinance can still be encountered anywhere on the dunes. The dunes were first opened to vehicle traffic in 1916 when a road made of wooden boards was constructed to facilitate transportation between San Diego and Yuma. A short section of this “plank road” is preserved near the Grays Well Road exit on Interstate 8. A paved road replaced the plank road 10 years later, which was in turn replaced by Interstate 8, a modern four lane divided highway. However, the dunes proved to be an impassible barrier for the Southern Pacific Railroad (now the Union Pacific Railroad). The rail line west from Yuma, Arizona turns north toward Los Angles and runs the length of the dunes eastern edge. Drainage from the Chocolate and Cargo Muchacho Mountains is now channelized by a series of V shaped berms that funnel the runoff under the tracks through large culverts or bridges. In recent decades, the Algodones Dunes has become one of the premiere sites for off road vehicle recreation in California. Crowds on winter holiday weekends may approach 150,000 people. The traffic from these vehicles has largely denuded the open sand habitats south of Highway 78. This traffic largely disappears as the weather warms and the peak season for insect activity approaches. The latest human modification of the dunes is the construction of a border wall along the California–Mexican border. The border wall is a 15-foot high metal fence with an undeveloped sand road for the use of U.S. Immigration running along the wall on the United States side.

Many of these human alterations of the dunes environment have probably had little effect on the dunes insect fauna; others have caused significant alterations. The two developments that probably caused the most significant alterations to the insect fauna are the agricultural development of the Imperial Valley and the building of the agricultural canals. Spring collections are dominated by aphids and their parasitoids during what must be an annual migration. Aquatic insects can be collected at nearly any time of the year at light traps or Malaise traps.

The eastern and western edges of the dunes substantially differ geologically and biologically. The drier western side is dominated by open dune habitats (Fig. 2b), psammophytic scrub (Fig. 2c) and creosote bush scrub (Fig. 2d). The eastern edge of the dunes is higher in elevation and benefits from run-off from the Chocolate and Cargo Muchacho Mountains, supporting more vegetation. Open and vegetated dunes, creosote bush scrub and gravel washes with microphyll woodland (Fig. 2e) characterize the eastern edge.

**Figure 3.**
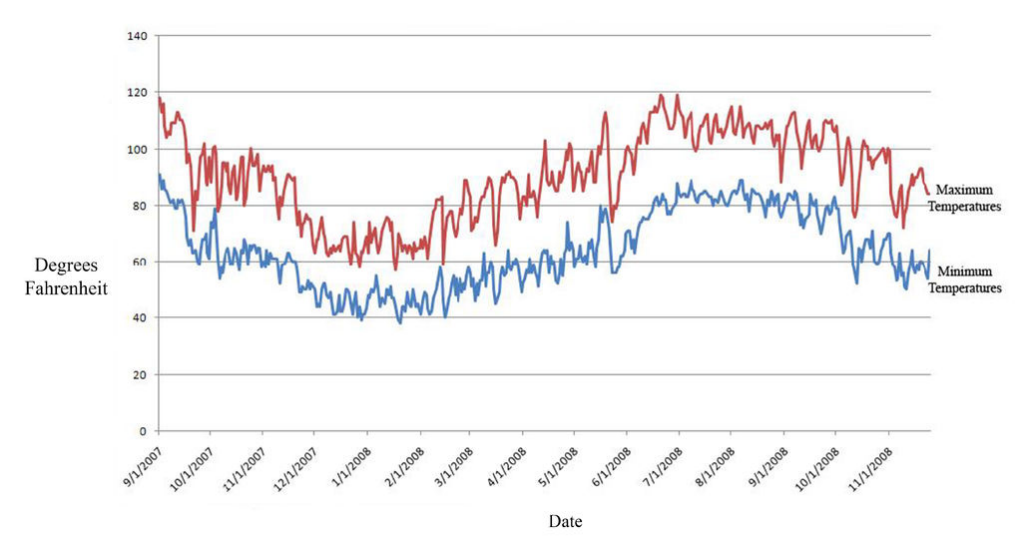
Algodones Dunes typical maximum and minimum air temperatures, data taken from the California Department of Water Resources Cahuilla and Buttercup weather stations from 2007 through 2008.

To live on the dunes, plants and animals must survive extreme temperatures, little rainfall and shifting substrates, comprising some of the harshest conditions in North America. Rainfall averages 76 mm annually, half falling during the winter, half in the summer monsoon. Prevailing winds shift seasonally, originating from the west in the fall, winter and spring months and from the southeast in the summer. Wind speeds, particularly during the summer, can reach 95 kph (60 mph). Temperatures approximately range from a nighttime low of 4°C (39.2°F) in the winter to a daytime high of 49°C (120.2°F) in June (Fig. 3).

The flora of the ISDRA seems depauperate relative to areas elsewhere in the Colorado Desert, but vertebrate surveys suggest a rich diversity (EIS-BLM 2003, Franzreb 1978). Historically, beetles constituted the best known insect diversity of the dunes. Several species of insects have been thought to be endemic to the Algodones Dunes, and the Center for Biological Diversity (Bond et al. 2004) petitioned the Secretary of the Interior to list 16 insect taxa known only from the Algodones Dunes as threatened or endangered pursuant to the Endangered Species Act. The taxa included in the petition are: two sand wasps, *Microbembex elegans* Griswold and *Stictiella villegasi* Bohart; two bees, *Perdita algodones* Timberlake and *P. glamis* Timberlake; one vespid wasp, *Euparagia* n. sp. (subsequently described as *E. unidentata* Carpenter & Kimsey); two velvet ants, *Dasymutilla nocturna* Mickel and *Dasymutilla imperialis* Manley and Pitts; three jewel beetles, *Lepismadora algodones* Velten, *Prasinalia imperialis* (Barr), and *Agrilus harenus* Nelson; two scarab beetles, *Anomala hardyorum* Potts and *Cyclocephala wandae* Hardy; and four dune weevils, *Trigonoscuta rothi rothi* Pierce, *T. r. algodones* Pierce, *T. r. imperialis* Pierce, and *T. r. punctata* Pierce.

Thus, we sought to enumerate insect species diversity, expand the list of potentially endemic insects, characterize the systematic diversity, and parse the frequency distribution of insect life history traits in the Algodones Dunes. Accordingly, we periodically sampled diverse locations in the dunes (Fig. 1) using a variety of insect traps, aerial net collecting and sand-sifting to enhance the diversity of our capture. Ultimately we sought to develop a comprehensive understanding of the insect diversity of this region.

## Study Site

We surveyed the insects of the Imperial Sand Dunes Recreation Area from September 2007 until March 2016, making 383 collections during 22 collecting trips ranging in duration from 2 to 8 days. During the first year of the study, we repeatedly sampled five sites for five consecutive days during different seasons of the year for a seasonal faunal comparison. These sites were located in the three major vegetation types found on the dunes, psammophytic scrub, creosote bush scrub and microphyll woodland. Starting in the second year of the study and continuing into the third year, we sampled eight sites (four pairs) to compare faunal diversity between non-disturbed and badly disturbed habitats. This study has yet to be published. The four pairs were distributed approximately 0.5-2.4 km (0.03-1.5 mi) apart along Highway 78 from the Cahuilla Ranger Station on the west to Glamis on the east. One member of each pair was located approximately 0.3-1.6 km (0.2-1.0 mi) north of Highway 78 in the undisturbed North Algodones Dunes Wilderness and the other member a comparable distance south of Highway 78 in disturbed habitat heavily impacted by off-highway vehicles (Fig. 2f). In addition to the seasonal and disturbance comparison collections just enumerated, we serendipitously made an additional 323 collections throughout the dunes. These collections were made to take advantage of somewhat different vegetation or soil types, the availability of pools of water following rains, or of a particular plant species in bloom. The number of collecting localities is considerably less than the number of collections because some specific localities were visited on several collecting trips, resulting in multiple collection numbers for the same locality, and because some collections were made in such close proximity to each other that they are essentially from the same locality. If the geographic coordinates of all 383 collections we made are rounded to two decimal places, the collections are grouped into 103 rectangular areas approximately 935 m wide (the east-west direction) and 1109 m high (north-south direction). Such rectangles have an area of approximately 1.04 km^2^. Alternatively, if the coordinates are rounded to three decimal places, then 208 rectangular areas approximately 93.5 m wide and 110.9 m high with an area of approximately 10,400 m^2^ each are identified. Depending upon the degree of precision desired, the number of different collecting localities or sites can therefore be considered to be 103 or 208.

## Methods

We employed a variety of collecting techniques to sample insect diversity. Simple aerial net collecting, sand sifting through 0.3 cm (0.125 in) mesh screen, and hand-capture by team members supplemented Malaise, black light, pitfall, yellow bowl, blue vane and McPhail traps. Standard Malaise traps continuously collected flying and walking insects into catcher heads filled with propylene glycol or 95% ethanol or a combination of both (Fig. 4a). Black light traps consisted of a 12 VDC powered 20w black light tube horizontally fitted to vanes above a funnel and bucket arrangement to passively collect insects attracted to the UV light at night (Fig. 4a). Yellow bowl traps were filled with soapy water or propylene glycol. One-pint pitfall traps, filled with 1 inch of propylene glycol and set into the substrate under an elevated cover were used to capture insects seeking shelter during the day or walking into them at night. We additionally deployed blue vane and blue tube traps effective for collecting bees (Stephen and Rao 2005) (Fig. 4b). McPhail traps baited with carrion (chicken or rabbit parts), bananas, or acetic acid were deployed to capture insects attracted to these substances (Fig. 4b).

To sample fauna associated with each season, we attempted five-day-long sampling excursions to the dunes during the first year of the study, scheduling each in a different season insofar as was possible. During these excursions we set one Malaise trap, ten yellow bowls and ten pitfall traps, and ran a black light trap for one or two nights in each of the five season-comparison sites. Moderate to full gale force winds and sand storms sometimes blew equipment down or away, obliterating samples. As resources and opportunity permitted during these excursions, we also set out these devices in secondary non-permanent sites as well.

To sample fauna in disturbed and undisturbed habitats in the disturbance comparison locations along Highway 78, we set out eight black light traps simultaneously on one night, and eight Malaise traps continuously for five days in June, August and September 2009 and March 2010.

In later years of the study, sampling was primarily by hand netting and Malaise and black light traps.

**Taxonomic coverage.** We have developed a checklist of the insects of the Algodones Dunes, which is available on the Bohart Museum of Entomology website - http://bohart.ucdavis.edu/research.html. A more detailed database to manage all our insect specimen data is under construction but is not yet available on-line. Numerous experts in the systematic community assisted us with the identification of certain insect groups, particularly scientists at the California Department of Food & Agriculture, Plant Pest Diagnostics Branch; the University of California Davis; and the U.S. National Museum (see acknowledgments). This process of species identification remains on-going, and we have not identified some taxonomic groups where expertise remains absent, for example muscoid and nematoceran fly families, Trichoptera and Psocodea. Thus, the checklist and database remain incomplete for the taxa where identification remains on-going. Specimens are deposited in the Bohart Museum of Entomology, University of California, Davis.

Temperature, rainfall and wind speed/direction were taken from the California Department of Water Resources Cahuilla (http://cdec.water.ca.gov/cgi-progs/queryF?CAU) and Buttercup (http://cdec.water.ca.gov/cgi-progs/queryF?BUT) weather stations, located at the middle and southern end of the dunes respectively.

## Results

Our nine-year survey of the insect fauna yielded five major observations. 1) The order Hymenoptera constituted the most diverse order comprising more than 45% of the insect species sampled and 42% of the total animal species recorded on the Algodones Dunes. 2) At least 79 species (roughly 4%) have thus far only been recorded from the dunes. 3) Insect species diversity and abundance peaked during the hottest part of the year between the months of May and September. 4) The insect fauna seems dominated by parasitoids and herbivores. 5) Large numbers of aquatic and agricultural pest insects that cannot survive on the dunes disperse there, likely from the Coachella and All-American Canals or from agricultural lands to the west.

**Species Composition.** The insect fauna of the Algodones Dunes is unexpectedly large, with 1,840 species in 21 orders and 244 families identified thus far (Table 1). Published records of beetle and spider wasps (Pompilidae) species collected in the dunes suggest that for these groups at least we have found only about 42% of the species previously recorded from the dunes (Table 2). Thus, realistically the insect fauna may be much greater than 2,000 species.

**Figure 5.**
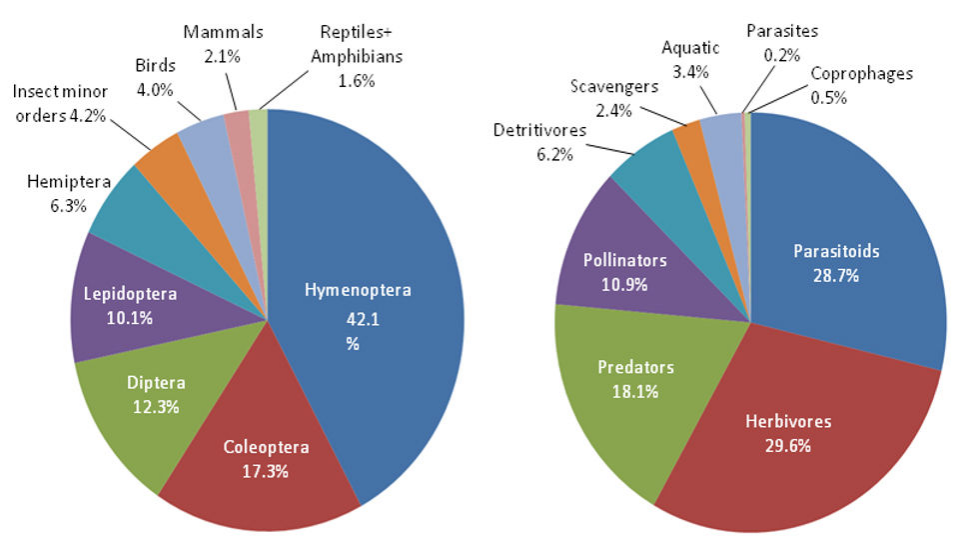
Pie charts showing proportions of life history traits (right) and the relative proportions of each animal group and insect orders (left).

With the largest number of species, 42.1%, the order Hymenoptera dominates this habitat, followed by Coleoptera 17.3%, Diptera 12.3%, Lepidoptera (10.1%) and Hemiptera 6.3% (Fig. 5). We additionally characterized biological diversity among these insect groups by scoring species into 8 larval life history types including: coprophages, detritivores/fungivores, herbivores (including foliage, wood and seed feeders), parasites, parasitoids, pollen/nectar feeders, predators, and scavengers (defined as feeding on dead animals). Larval parasites rarely kill their hosts differentiating them from parasitoid larvae which generally feed on and ultimately kill their hosts as they mature. Herein we scored such species as either parasites or parasitoids according to what is known for the larval habits for the family or genus. Larvae from aquatic habitats were separately treated as a habitat-specific category. Mosquitoes are scored as aquatic even though the adult females are parasites. The largest numbers of species on the dunes are herbivores (29.6%), followed by parasitoids (28.7%), predators (18.1%), pollen/nectar feeders (10.9%), detritivores (6.2%), scavengers (2.4%), aquatic (3.4%), parasites (0.2%), and coprophages (0.5%) (Fig. 5).

**Potentially Endemic Species.** We compiled a list of 79 species of insects recorded only from the dunes including those new to science and a number of described species (Table 3). These represent about 4% of the species collected to date, and the majority of these occur in the open dunes and psammophytic scrub habitats. The psammophytic scrub habitat includes sparse, scattered perennial plants, exemplified by *Eriogonum*, *Astragalus*, *Croton, Helianthus* and *Tiquilia*, and a diversity of short-lived annuals. For example, the endemic predatory wasp *Euparagia unidentata* Carpenter & Kimsey (Vespidae) seems closely associated with *Croton*, one of the few sources of summer nectar in the dunes. *Microbembix elegans* Griswold (Crabronidae) adults visit *Croton* and *Eriogonum* for nectar but feed their larvae dead insects scavenged on the sand. Two species of endemic Jewel Beetle, *Lepismadora algodones* Velten and *Acmaeroderoides stramineus* Nelson (Buprestidae) apparently use *Tiquilia* as a larval host plant. Interestingly, we collected a large number of potentially endemic species on the woody shrubs along Gecko Road, a region on the west side of the dunes heavily impacted by visitors using off-road vehicles during the winter months.

**Seasonality.** Adult insect diversity and abundance peaked during the hottest part of the year, between the months of May and September. Daytime temperatures during this period ranged from 32-49°C (90-120.2°F), with the highest temperatures usually occurring in June (Fig. 3). Sand surface temperatures commonly climbed at least as high as 65.5°C (150°F). Although rainfall typically averages 76 mm per year, the amount was closer to 53-56 mm per year during our study. We observed a temporary amplification of insect numbers and species several weeks after monsoonal rainfall during the summer.

Species of insects native to the dunes clearly predominated in summer months, when exotic species infrequently occurred. However, during the cool winter months bean aphid (*Aphis fabae* Scopoli) and pea aphid (*Acyrthosiphon pisum* Mordvilko) (Aphididae) and a variety of pest noctuid moths including cutworms and army worms (Noctuidae) exemplified the exotic species that dominated the insect fauna on the dunes. These likely dispersed from adjacent agricultural land to the west. Winged adult bean and pea aphids but no wingless offspring, together with adults and nymphs of *Acyrthosiphon kondoi* Shinji, fed on the endangered Peirson’s Milk Vetch in March. Thus it seems unlikely that bean or pea aphids reproduce on this plant.

**Scavengers and Fungivores.** The native insect fauna contains a large number of scavengers and fungivores. These insects do not constitute the most species-rich taxa, but dominate in numbers of individuals. Predominant scavengers on the dunes include the Algodones sandtreader, *Macrobaenetes algodonensis* Tinkham (Rhaphidophoridae), two species of *Microbembix elegans* and *argyropleura* Bohart (Crabronidae), and a diversity of tenebrionid beetle species (Coleoptera). Fungivores, particularly larvae of fungivorous beetles, for example Anthicidae, seem to take advantage of a perennially moist subsurface stratum where mycorrhizal and decomposing fungi grow. A single, one-night black light trap sample in June 2010, when temperatures were high and water availability was low, contained approximately 22,000 individuals of the anthicid beetle, *Anthicus cervinus* LaFerté-Sénectère (Anthicidae).

## Dunes exotics

Numerous exotic crop or horticultural pest insects that are unlikely to survive for long on the dunes, constituting about 2% of species, land on the dunes, most likely after dispersion from adjacent western agricultural lands. These include the aphid species that feed primarily on alfalfa, and *Aphis nerii* Boyer de Fonscolombe that feeds on milkweed (*Asclepias*).

Interestingly, aquatic insects, constituting over 3% of the species collected, must originate from one of the canals, guzzlers or railroad irrigation puddles, or indirectly from the Colorado River to the east and southeast. Aquatic insects often disperse into adjacent desert regions where they cannot survive, but provide food resources to local predators and scavengers (Jackson and Fisher 1986). For example we collected 10 species of dragonflies and damselflies where no standing water existed. Seven species of mosquitoes, *Anopheles freeborni* Aitken, *Culex quinquefasciatus* Say, *Culex tarsalis* Coq., *Culiseta incidens* (Thomson), *Culiseta inornata* (Williston), *Psorophora signipennis* (Coq.) and *Psorophora toltecum* (Dyar & Knab) were collected, primarily in black light traps. We also found one species of sisyrid spongilla fly (*Climacea californica* Chandler; Neuroptera), numerous chironomid midges, four species of Notonectidae, one species of Corixidae and five species each of Trichoptera and Ephemeroptera. Those that occupy moving water habitats likely originated from the Coachella or All-American Canals.

We also occasionally observed exotic species appearing only once on the dunes and then disappearing. In 2007 we recorded large numbers of two species of earwig, *Euborellia cincticollis* (Gerstaecker) () and *Labidura riparia* (Pallas) (Labiduridae) at several localities. In the same year the house cricket, *Acheta domesticus* L. (Gryllidae) commonly occurred and additionally was observed in the nearby community of El Centro. We did not subsequently collect these species on the dunes.

A component of the insect fauna that cannot derive from the dunes and yet are not chance immigrants from local agricultural or aquatic habitats include known migrating species, such as painted lady butterflies, *Vanessa cardui* (L.) (Nymphalidae), and two dragonfly species, *Sympetrum corruptum* (Hagen) (Libellulidae) and *Aeshna junius* Say (Aeshnidae).

Long range aerial plankton would also appear to land/fall on the dunes. For example, we recorded two specimens of *Amphidecius schickae* Heydon (Pteromalidae) from the dunes; the only known hosts of this species, *Neuroderus* sp. (Cynipidae), feed on oaks, yet the nearest oaks to the dunes are approximately 100 km to the west. One specimen of the *Aristolochia* pipevine specialist *Battus philenor* (L.) (Papilionidae) was also found on the open dunes nearly 100 km from the closest known source of native *Aristolochia* in western Arizona.

## Conclusions

Species in the order Hymenoptera clearly dominate this sand dune habitat. Even if expanded sampling techniques added Coleoptera species, the number of species would have to double to approach the number of Hymenoptera. Such numbers make sense; more than half of Hymenoptera collected were parasitoids of other insects or non-insect arthropods. Additionally, given the large number of fungivorous insects in our samples fungi must be a major yet cryptic presence in these dunes.

## Figures and Tables

**Figure 2a.**
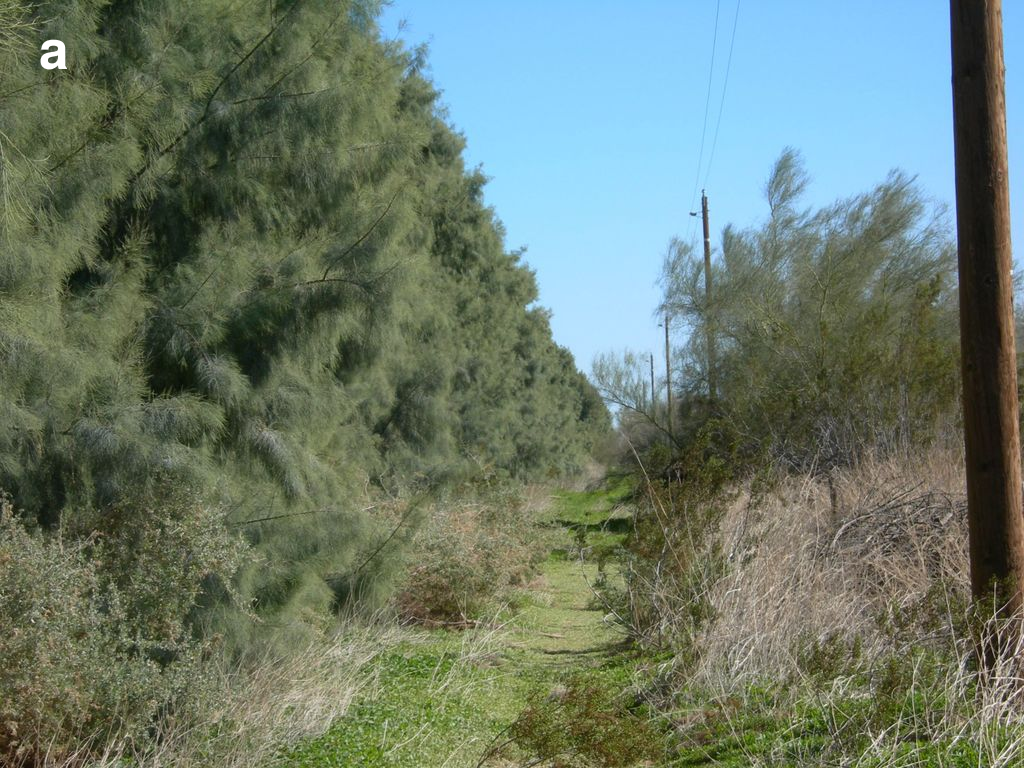
Tamarisk wind break planted by the Union Pacific Railroad on left.

**Figure 2b.**
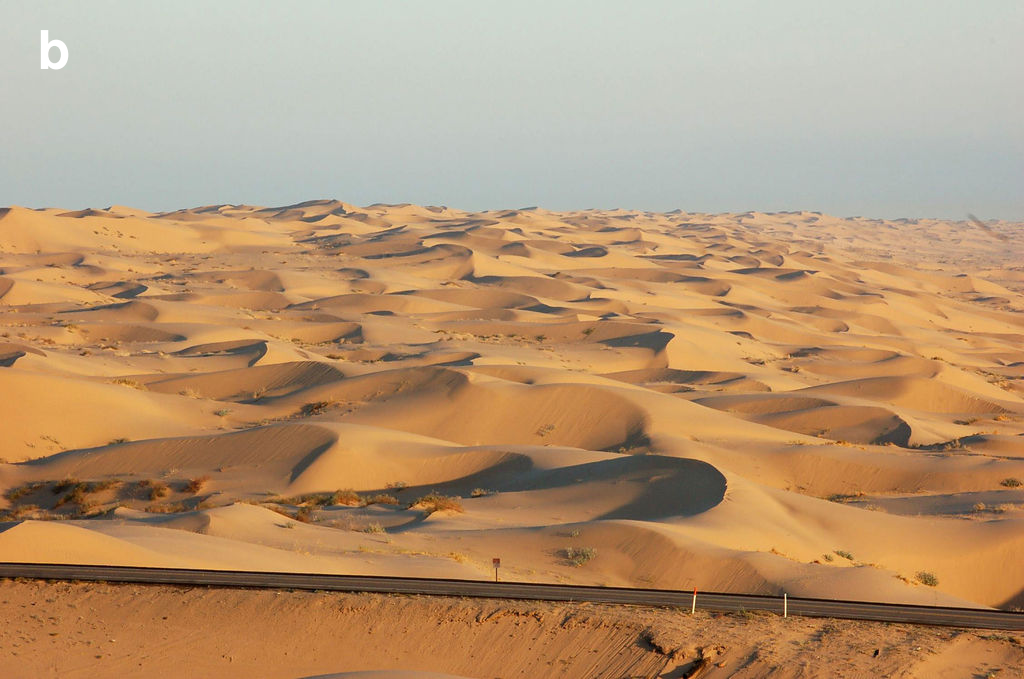
Open dunes looking south.

**Figure 2c.**
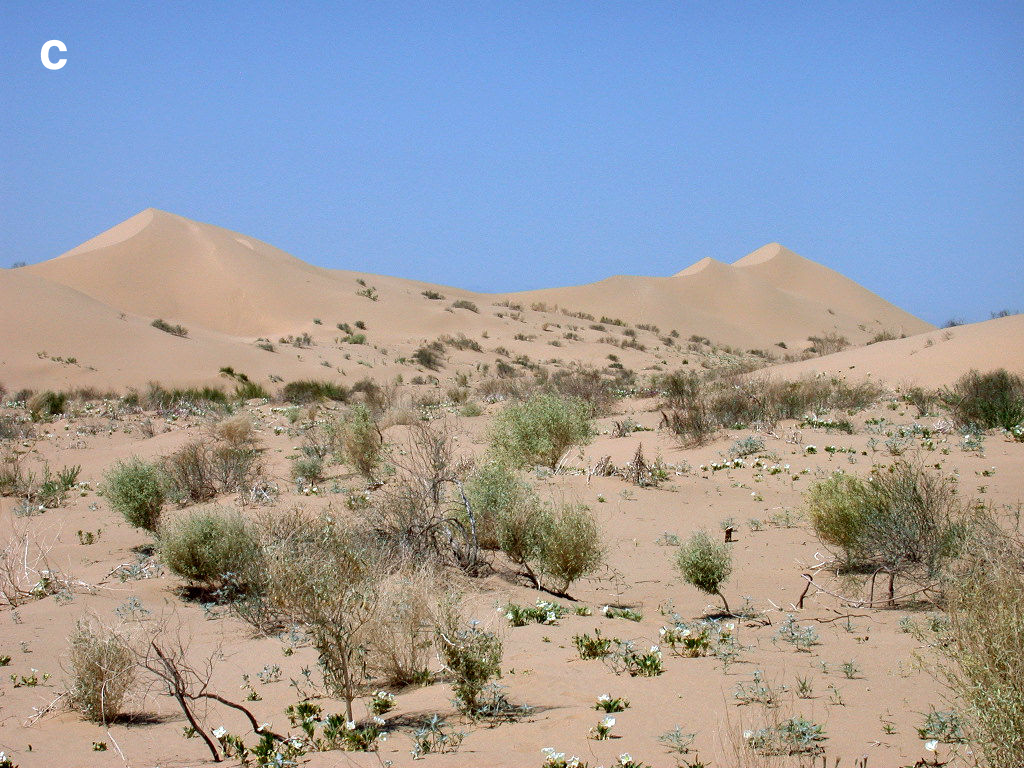
Psammophytic scrub.

**Figure 2d.**
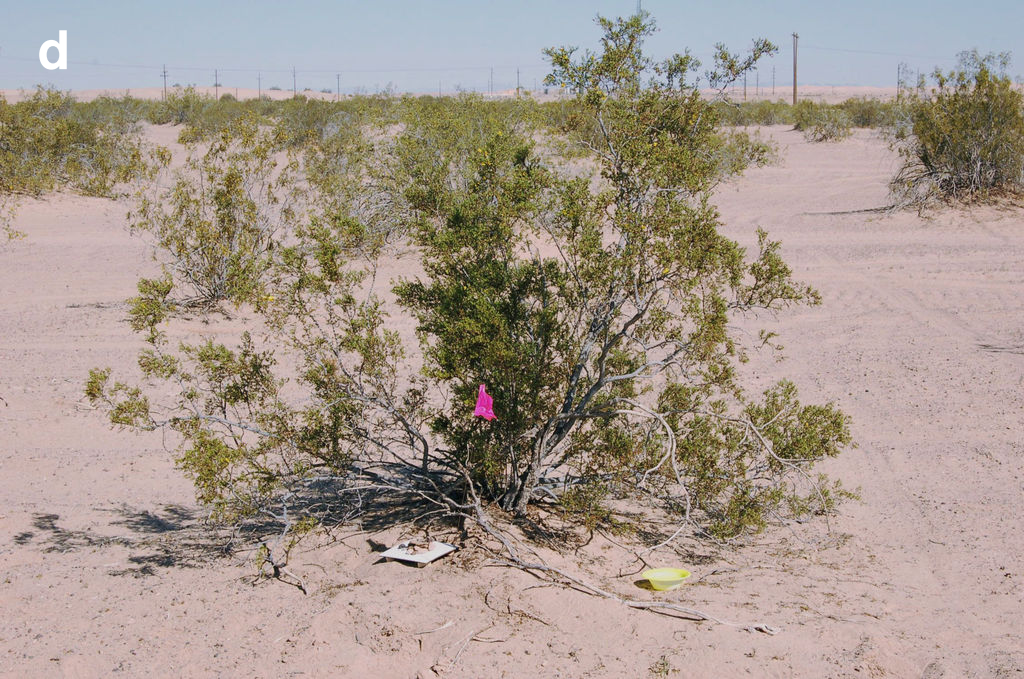
Creosote bush scrub.

**Figure 2e.**
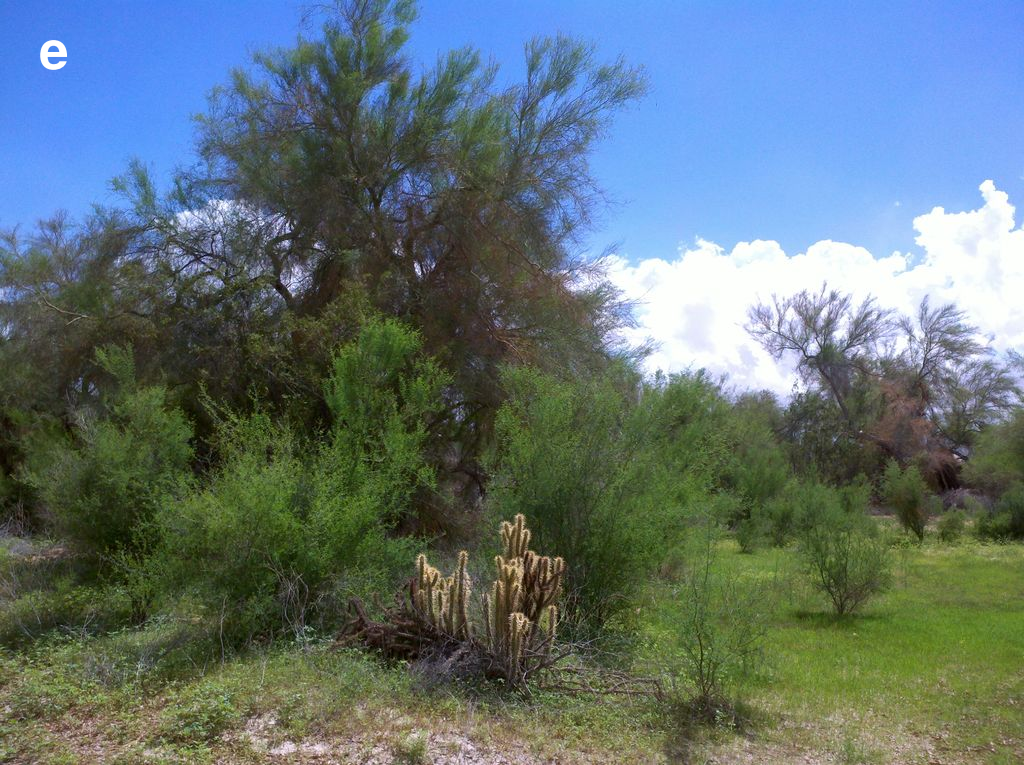
Microphyll woodland.

**Figure 2f.**
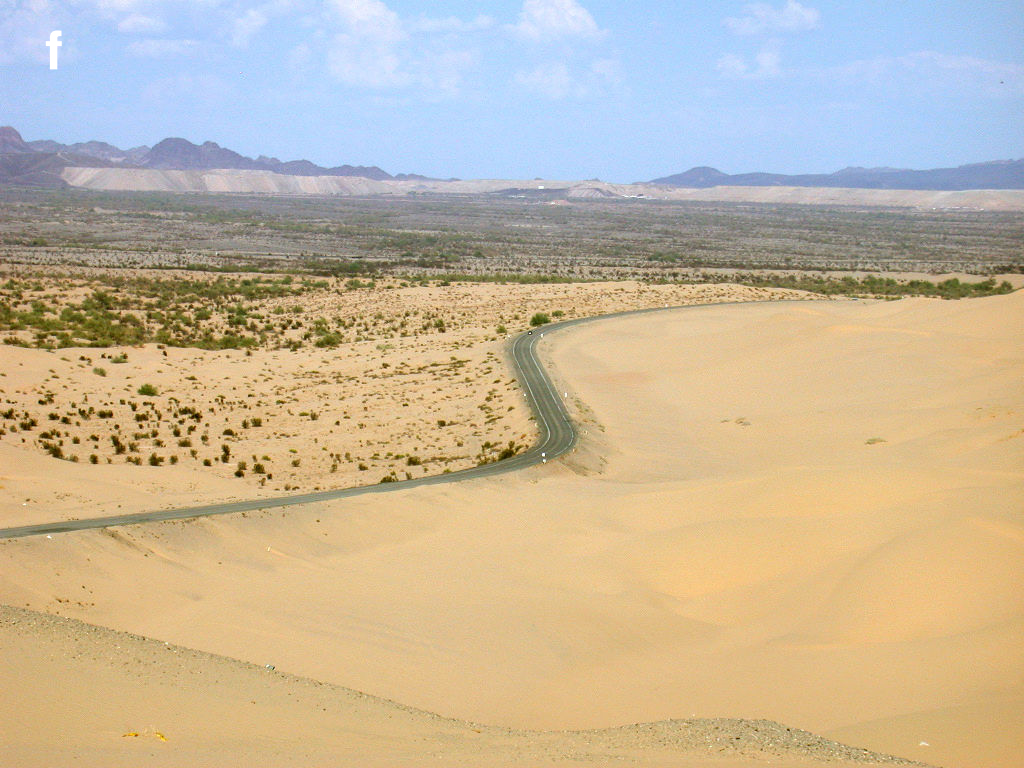
Off-road impact on south side of highway 78.

**Figure 4a.**
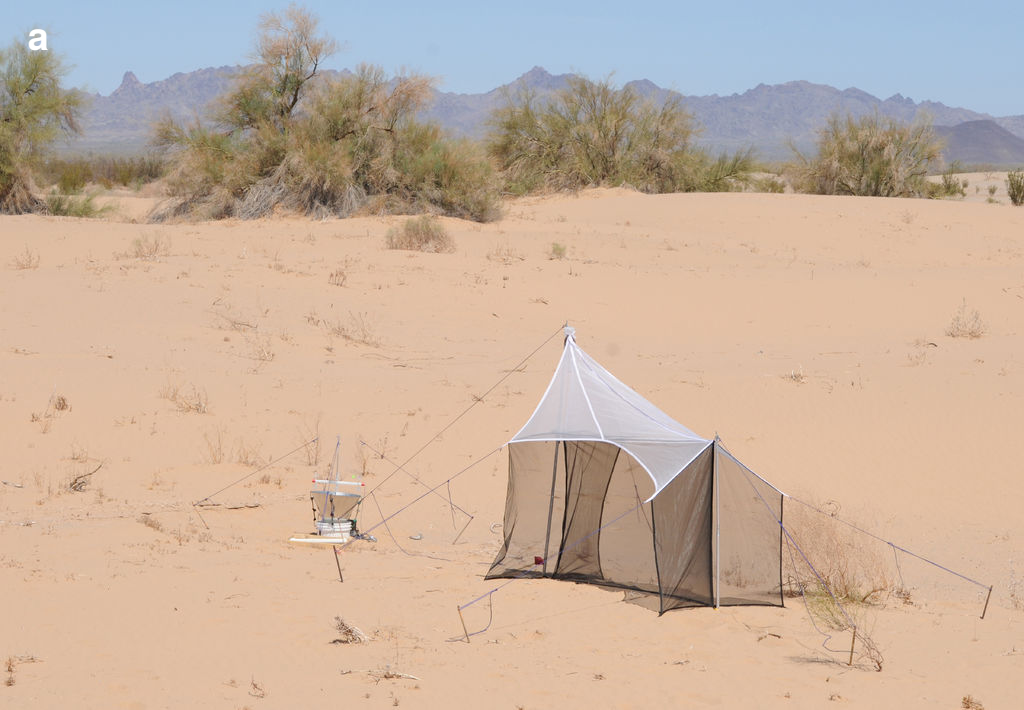
Blacklight and malaise traps.

**Figure 4b.**
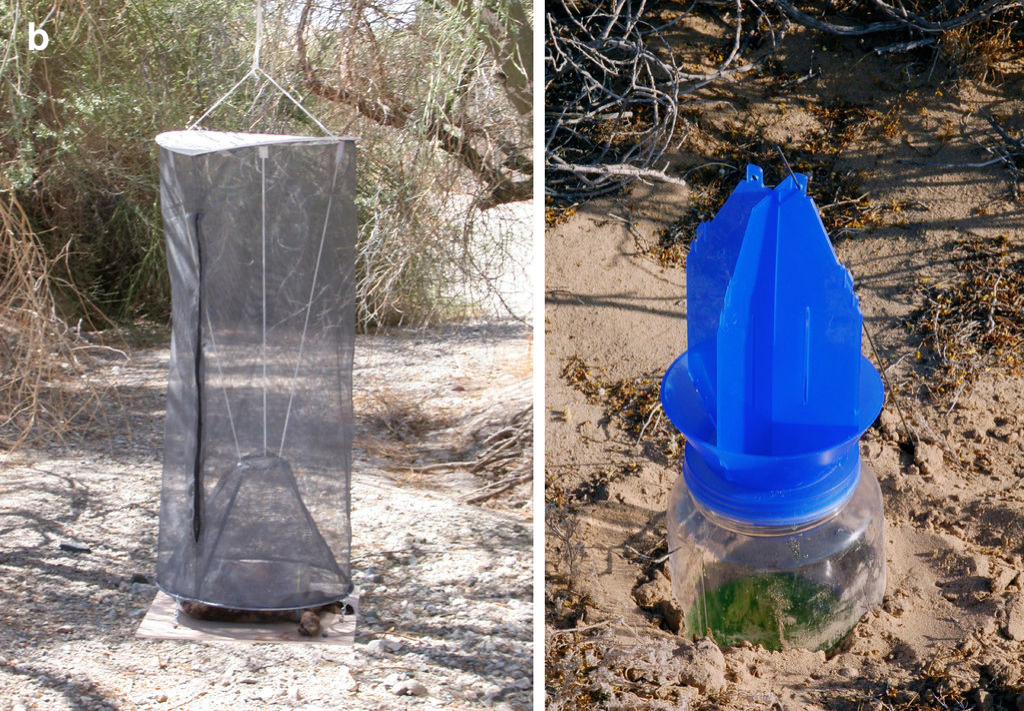
McPhail and blue vane traps.

**Table 1.** Overview of the insects collected on the Algodones Dunes, Imperial County, California between 2007 and 2016.

Order	Total number of families	Total number of species	Number of species known only from the dunes	Total number of exotic species*	Total aquatic species
Blattodea	2	3	0	1	0
Coleoptera	54	343	17	9	15
Dermaptera	2	2	0	2	0
Diptera	50	244	7	4	20
Embiidina	1	1	0	0	0
Ephemeroptera	1	5	0	0	5
Hemiptera	32	131	0	8	6
Hymenoptera	45	837	52	2	0
Isoptera	2	3	0	0	0
Lepidoptera	28	200	0	6	2
Mantodea	1	2	0	0	0
Microcoryphia	1	1	0	0	0
Neuroptera	5	31	0	0	1
Odonata	6	10	0	0	10
Orthoptera	4	11	1	1	0
Phasmida	1	1	0	0	0
Psocodea	1	5	0	0	0
Strepsiptera	1	1	0	0	0
Thysanoptera	2	2	0	1	0
Trichoptera	3	5	0	0	5
Zygentoma	2	2	0	0	0
Total	244	1840	77	34	64

*Insects exotic to North America.

**Table 2.** Comparison of species recorded from the Algodones Dunes in the literature versus those collected in the current study, and those found in both. Literature records are from: ^1^Andrews et al. 1979; ^2^Wasbauer and Kimsey 2010; ^3^Rolf Albuu, pers. comm.

Taxon	Number of published species	Number of survey species	Difference	Number of species in both	% of species in both
** Hymenoptera **					
*Aporinellus* (Pompilidae)^2^	6	4	2	2	25
*Arachnospila* (Pompilidae)^2^	7	2	5	1	13
*Episyron* (Pompilidae)^2^	3	1	2	1	33
*Pompilus* (Pompilidae)^2^	3	2	1	1	25
** Coleoptera **					
*Asbolus* (Tenebrionidae)^3^	4	2	2	2	50
*Chilometopon* (Tenebrionidae)^3^	5	3	2	3	60
*Cryptoglossa* (Tenebrionidae)^1^	2	1	1	1	50
*Cymatodora* (Cleridae)^1^	3	1	2	1	33
*Diplotaxis* (Scarabaeidae)^1^	6	0	6	0	0
*Eustattus* (Tenebrionidae)^3^	5	3	2	3	60
*Glaresis* (Glaresidae)^1^	3	0	3	0	0
*Horistonotus* (Elateridae)^3^	0	2	2	0	0
*Tricorynus* (Anobiidae)^1^	3	0	3	0	0

**Table 3.** Insect species only recorded from the Algodones Dunes as of the current study. (*Taxa newly recognized during this study.)

	** Coleoptera **	
1	*Acmaeroderoides stramineus* Nelson	Buprestidae
2	*Agrilus harenus* Nelson	Buprestidae
3	*Lepismadora algodones* Velten	Buprestidae
4	*Prasinalia imperialis* (Barr)	Buprestidae
5	*Hyperaspidius algodones* Gordon	Coccinellidae
6	*Trigonoscuta rothi rothi* Pierce	Curculionidae
7	*Trigonoscuta rothi algodones* Pierce	Curculionidae
8	*Trigonoscuta rothi imperialis* Pierce	Curculionidae
9	*Trigonoscuta rothi punctata* Pierce	Curculionidae
10	*Horistonotus* n. sp. 1*	Elateridae
11	*Horistonotus* n. sp. 2*	Elateridae
12	*Anomala carlsoni* Hardy	Scarabaeidae
13	*Anomala hardyorum* Potts	Scarabaeidae
14	Cyclocephala wandae Hardy	Scarabaeidae
15	*Edrotes arens* La Rivers	Tenebrionidae
16	*Eusattus dilatatus* LeConte	Tenebrionidae
17	*Nocibiotes crassipes* (Casey)	Tenebrionidae
18	*Tonibius sulcatus* (Casey)	Tenebrionidae
	** Diptera **	
19	*Apiocera warner* Cazier	Apioceridae
20	*Efferia macroxipha* Forbes	Asilidae
21	*Elachiptera* n. sp.*	Chloropidae
22	*Trixoscelis* n. sp.*	Heleomyzidae
23	Blaesoxipha (Acanthodotheca) n. sp.*	Sarcophagidae
24	*Eumacronychia* n. sp. 1*	Sarcophagidae
25	*Eumacronychia* n. sp. 2*	Sarcophagidae
	** Hymenoptera **	
26	*Perdita algodones* Timberlake	Andrenidae
27	*Perdita glamis* Timberlake	Andrenidae
28	*Psilochalcis* n. sp. 1	Chalcididae
29	*Psilochalcis* n. sp. 2	Chalcididae
30	*Psilochalcis* n. sp. 3	Chalcididae
31	*Psilochalcis* n. sp. 4	Chalcididae
32	*Microbembex elegans* Griswold	Crabronidae
33	*Plenoculus* n. sp. 1*	Crabronidae
34	*Plenoculus* n. sp. 2*	Crabronidae
35	*Plenoculus* n. sp. 3*	Crabronidae
36	*Plenoculus* n. sp. 4*	Crabronidae
37	*Plenoculus* n. sp. 5*	Crabronidae
38	*Solierella* n. sp.*	Crabronidae
39	*Stictiella villegasi* Bohart	Crabronidae
40	*Banacuniculus dis* Buffington	Eucoilidae
41	*Ganaspidium* n. sp.*	Eucoilidae
42	*Tenuipetiolus* n. sp.*	Eurytomidae
43	*Dasymutilla imperialis* Manley & Pitts	Mutillidae
44	*Dasymutilla nocturna* Mickel	Mutillidae
45	*Sphaerophthalma ecarinata* Schuster	Mutillidae
46	*Sphaerophthalma django* Pitts & Wilson*	Mutillidae
47	*Ageniella arenicola* Wasbauer & Kimsey*	Pompilidae
48	*Ageniella pernia* Wasbauer & Kimsey*	Pompilidae
49	*Acordulecera algodones* Smith	Pergidae
50	*Acordulecera kimseyi* Smith	Pergidae
51	*Caenocrepis* n. sp.*	Pteromalidae
52	*Catalaccus* n. sp. 1*	Pteromalidae
53	*Catalaccus* n. sp. 2*	Pteromalidae
54	*Chlorocytus* n. sp.*	Pteromalidae
55	*Epipteromalus* n. sp. 1*	Pteromalidae
56	*Epipteromalus* n. sp. 2*	Pteromalidae
57	*Epipteromalus* n. sp. 3*	Pteromalidae
58	*Gastrancistrus* n. sp. 1*	Pteromalidae
59	*Gastrancistrus* n. sp. 2*	Pteromalidae
60	*Gastrancistrus* n. sp. 3*	Pteromalidae
61	*Gastrancistrus* n. sp. 4*	Pteromalidae
62	Gastrancistrus n. sp. 5*	Pteromalidae
63	Gastrancistrus n. sp. 6*	Pteromalidae
64	*Halticoptera* n. sp.*	Pteromalidae
65	*Heteroschema* n. sp.*	Pteromalidae
66	*Lyrcus* n. sp. 1*	Pteromalidae
67	*Lyrcus* n. sp. 2*	Pteromalidae
68	*Lyrcus* n. sp. 3*	Pteromalidae
69	*Lyrcus* n. sp. 4*	Pteromalidae
70	*Lyrcus* n. sp. 5*	Pteromalidae
71	*Pachyneuron* n. sp.*	Pteromalidae
72	*Pteromalus* sp. 1*	Pteromalidae
73	*Pteromalus* sp. 2*	Pteromalidae
74	*Pteromalus* sp. 3*	Pteromalidae
75	*Pteromalus* sp. 4*	Pteromalidae
76	*Sedomaya glamisensis* Kimsey & Wasbauer	Tiphiidae
77	*Pseuderimerus* n. sp.*	Torymidae
78	*Euparagia unidentata* Carpenter & Kimsey*	Vespidae
	** Orthoptera **	
79	*Macrobaenetes algodonensis* Tinkham	
